# Assessment of Vedolizumab Disease‐Drug‐Drug Interaction Potential in Patients With Inflammatory Bowel Diseases

**DOI:** 10.1002/cpdd.891

**Published:** 2020-12-16

**Authors:** Wan Sun, Richard A. Lirio, Jennifer Schneider, Jiri Aubrecht, Harisha Kadali, Mike Baratta, Parul Gulati, Ajit Suri, Tiffany Lin, Raghavan Vasudevan, Maria Rosario

**Affiliations:** ^1^ Takeda Cambridge Massachusetts USA; ^2^ Certara USA, Inc. Princeton New Jersey USA

**Keywords:** biologics, clinical pharmacology, disease management, drug‐drug interactions, gastrointestinal

## Abstract

Disease‐drug‐drug interactions (DDDIs) have been identified in some inflammatory diseases in which elevated proinflammatory cytokines can downregulate the expression of cytochrome P450 (CYP) enzymes, potentially increasing systemic exposure to drugs metabolized by CYPs. Following anti‐inflammatory treatments, CYP expression may return to normal, resulting in reduced drug exposure and diminished clinical efficacy. Vedolizumab has a well‐established positive benefit‐risk profile in patients with ulcerative colitis (UC) or Crohn's disease (CD) and has no known systemic immunosuppressive activity. A stepwise assessment was conducted to evaluate the DDDI potential of vedolizumab to impact exposure to drugs metabolized by CYP3A through cytokine modulation. First, a review of published data revealed that patients with UC or CD have elevated cytokine concentrations relative to healthy subjects; however, these concentrations remained below those reported to impact CYP expression. Exposure to drugs metabolized via CYP3A also appeared comparable between patients and healthy subjects. Second, serum samples from patients with UC or CD who received vedolizumab for 52 weeks were analyzed and compared with healthy subjects. Cytokine concentrations and the 4β‐hydroxycholesterol‐to‐cholesterol ratio, an endogenous CYP3A4 biomarker, were comparable between healthy subjects and patients both before and during vedolizumab treatment. Finally, a medical review of postmarketing DDDI cases related to vedolizumab from the past 6 years was conducted and did not show evidence of any true DDDIs. Our study demonstrated the lack of clinically meaningful effects of disease or vedolizumab treatment on the exposure to drugs metabolized via CYP3A through cytokine modulation in patients with UC or CD.

Disease‐drug‐drug interactions (DDDIs) have been identified during altered immunological states such as infection and inflammation.^1^, ^2^ Several inflammatory diseases are associated with decreased expression and/or activity of specific cytochrome P450 (CYP) enzymes such as CYP3A that are involved in hepatic drug clearance, potentially increasing the systemic exposure of drug substrates metabolized by CYPs and possibly resulting in increased incidence of adverse events.^2^, ^3^, ^4^ Pro‐ and anti‐inflammatory cytokines, such as interleukin (IL)‐1β, IL‐6, IL‐10, interferon (IFN)‐α or IFN‐β, and tumor necrosis factor (TNF)‐α, are known to influence and regulate hepatic CYP expression during inflammation.^2^, ^3^, ^4^ IL‐8 is also a recognized proinflammatory cytokine and is reported to have a high concentration in patients with inflammatory bowel disease (IBD); however, to date, its effect on CYP expression and/or activity is still unclear.^1^, ^5^, ^6^

Several biologic agents, such as cytokine‐modulating monoclonal antibodies (mAbs) used to treat inflammatory conditions, are also reported to alter the disposition of drugs that are metabolized by CYP enzymes.^2^, ^7^ In patients with a chronic inflammatory condition treated with an anti‐inflammatory drug (eg, anti‐TNF), CYP expression may become normalized, resulting in increased drug clearance, reduced drug exposure, and diminished clinical efficacy.^3^

IBDs, such as ulcerative colitis (UC) and Crohn's disease (CD), are chronic conditions conventionally treated with aminosalicylates, corticosteroids, and immunomodulators (IMMs). Patients with inadequate response or no response to these drugs are recommended to receive treatment with biologic agents such as anti‐TNF (eg, infliximab, adalimumab), anti‐integrin (eg, vedolizumab), and anti‐IL (eg, ustekinumab) therapies.^8^, ^9^ Corticosteroids such as prednisone, budesonide, and IMMs such as cyclosporine are partially or mostly metabolized by CYP3A enzymes.^1^ Hence, exposure to these drugs may be affected by IBD or treatment with biologic agents if expression or activity of CYP enzymes is significantly altered. Evaluation of the potential changes in the concentration or therapeutic effect of these CYP substrate drugs given concomitantly with biologic agents is recommended.^2^, ^10^

Vedolizumab is a humanized mAb that specifically binds to the human lymphocyte α_4_β_7_ integrin and acts as a gut‐selective anti‐inflammatory agent.^11^ Intravenous vedolizumab has been approved for the treatment of adult patients with moderately to severely active UC or CD. Given its gut‐selective mechanism, vedolizumab is not believed to have any systemic immunosuppressive activity and is not regarded as a cytokine modulator.^12^ Furthermore, vedolizumab is not believed to activate leukocytes and affect cytokine production by differentiated T lymphocytes.^11^, ^12^ These observations are consistent with extensive clinical and real‐world data demonstrating that vedolizumab is effective and generally safe for the treatment of patients with UC or CD.^13^, ^14^, ^15^, ^16^, ^17^, ^18^

Here, we aimed to confirm and extend the established safety profile of vedolizumab with respect to potential DDDIs. This study used a stepwise assessment to evaluate the DDDI potential of vedolizumab to affect exposure to CYP‐substrate drugs through modulation of inflammatory cytokines in patients with UC or CD.

## Methods

### Literature Data Review

A literature data review was conducted in the PubMed and EMBASE databases for reports published from 1995 to 2020. Data on serum cytokine concentrations and exposure to commonly used medications for UC or CD metabolized by CYP enzymes were collected from relevant publications and compared between healthy subjects and patients with UC or CD.

Baseline serum concentrations of the cytokines IL‐1β, IL‐6, IL‐8, IL‐10, and TNF‐α in patients with UC or CD and healthy subjects were collated from publications that reported quantitative measurements. Exposure to corticosteroids (eg, prednisolone, budesonide) or IMMs (eg, cyclosporine), assessed by pharmacokinetic (PK) parameters such as area under the curve (AUC) and maximum plasma concentration (C_max_) was reviewed in patients with UC or CD and compared with healthy subjects. Healthy subjects included those who had no organic disease or clinical signs of infection, were seeking medical attention for reasons other than intestinal inflammation or cancer or not receiving anti‐inflammatory treatment, and other healthy volunteers or individuals based on physical examination and laboratory tests.

## Cytokines and Endogenous CYP3A4 Biomarker Analysis

### Overall Study Design

A retrospective analysis was conducted to explore any potential effect of UC or CD and vedolizumab treatment on CYP expression and activity. Archived PK serum samples collected before and during the induction and maintenance phases of vedolizumab treatment in completed phase 3 trials with subcutaneous vedolizumab (VISIBLE 1 in UC [NCT02611830] and VISIBLE 2 in CD [NCT02611817]) were analyzed and compared with commercially procured samples from healthy subjects to evaluate the concentrations of selected cytokines and a surrogate biomarker of CYP3A4 activity. Healthy status was defined as no diagnosed hematologic or oncologic conditions, diabetes, autoimmune disorders, renal or liver impairment, or dementia or other significant mental illness based on physician assessment.

### Patients and Samples

The VISIBLE 1 and VISIBLE 2 studies were conducted to evaluate the efficacy and safety of subcutaneous vedolizumab as maintenance therapy in patients with moderately to severely active UC and CD, respectively. For both VISIBLE 1 (NCT02611830) and VISIBLE 2 (NCT02611817), the clinical study protocol and applicable protocol amendments, investigator's brochure, informed consent forms, and other study‐related documents were submitted to and approved by the local or central institutional review boards for all study sites. Both studies were conducted in compliance with the informed consent regulations stated in the Declaration of Helsinki, International Conference on Harmonisation Guidelines for Good Clinical Practice, and all applicable local laws and regulations. Separate ethics committee approval was not required for the current retrospective sample and data analyses.

In both studies, patients who achieved clinical response in week 6 following 2 doses of open‐label vedolizumab intravenous therapy in week 0 and week 2 and were randomized into the vedolizumab subcutaneous arm as maintenance treatment received vedolizumab 108 mg subcutaneously every 2 weeks beginning in week 6 through week 50. Both studies included a placebo control arm during the maintenance phase, and the VISIBLE 1 study also included a vedolizumab intravenous reference treatment arm during the maintenance phase.

Samples from patients with UC or CD selected for this retrospective analysis were collected at baseline and during the induction and maintenance phases of vedolizumab treatment. Sample selection was based on the collection date relative to the treatment periods. Samples within the established stability range of cytokines were preferentially selected over expired samples whenever possible. All samples were stored frozen at –70°C after collection and were shipped from the long‐term storage facility to predesignated bioanalytical laboratories.

### Study End Points

Cytokines were selected based on their reported impact on CYP expression. As IL‐1β, IL‐6, IL‐10, and TNF‐α are known to affect CYP expression,^2^, ^3^, ^4^ they are included in the analysis. Although the effect of IL‐8 on CYP expression is still unclear, high concentrations were reported in patients with IBD,^1^, ^5^, ^6^ and therefore also included. Although IFN‐γ was not reported to alter CYP activity, it was shown to be increased in patients with IBD.^6^, ^19^ Hence, it was included in this study as a reference cytokine to validate the assay platform and verify the active disease status of patients.

CYP3A4 is a predominant enzyme in the human liver and is involved in the metabolism of approximately 40% of therapeutic agents.^4^ Cholesterol is metabolized by CYP3A4 to 4β‐hydroxycholesterol, so the concentrations of both cholesterol and 4β‐hydroxycholesterol were measured in the samples. Considering the intrinsic variability in the concentrations of these endogenous markers, the ratio of 4β‐hydroxycholesterol to cholesterol (4β‐OHC/C) was used as a surrogate marker to assess induction or inhibition of CYP3A activity.^20^ If CYP3A4 becomes significantly suppressed in patients with active IBD, a lower ratio of 4β‐OHC/C for these patients relative to healthy subjects is expected to be observed. This ratio is expected to increase if CYP3A4 becomes normalized with vedolizumab treatment.

### Bioassays and Data Analysis

The concentrations of serum cytokines were measured using a validated MSD cytokine 10‐V‐Plex sandwich immunoassay by Q2 Solutions Laboratories (Valencia, California). The assay's quantification range was 1.3‐1060, 0.54‐498, 0.81‐743, 0.57‐498, 0.34‐315, and 0.34‐320 pg/mL for IFN‐γ, IL‐1β, IL‐6, IL‐8, IL‐10, and TNF‐α, respectively. Intra‐assay precision values ranged from 2.4% to 3.3% for IFN‐γ, 1.7% to 4.3% for IL‐1β, 3.4% to 6.3% for IL‐6, 1.2% to 4.6% for IL‐8, 1.6% to 5.3% for IL‐10, and 0.8% to 3.0% for TNF‐α, whereas interassay precision values ranged from 3.9% to 5.7% for IFN‐γ, 4.2% to 11.3% for IL‐1β, 3.2% to 6.1% for IL‐6, 3.8% to 6.1% for IL‐8, 2.5% to 4.6% for IL‐10, and 3.9% to 5.7% for TNF‐α. Interassay accuracy values ranged from –7.6 to –1.5 for IFN‐γ, –1.1 to 3.5 for IL‐1β, –0.7 to 8.1 for IL‐6, –10.6 to 6.3 for IL‐8, –4.5 to –2.6 for IL‐10, and –8.3 to 3.5 for TNF‐α.

Established stability range for cytokines in serum samples ranged from 19 to 24 months. For the analysis of cytokines IL‐1β, IL‐6, and IL‐10, the majority of samples from patients with UC were expired (older than 24 months) but were still reported because no decay was observed with stability testing up to 24 months. None of the samples from patients with CD exceeded 24 months and therefore were all reported. In the case of cytokines IL‐8, IFN‐γ, and TNF‐α, all baseline and induction samples from patients with UC were expired (older than 19 months) and were not reported because decay was observed in serum samples beyond 19 months. Most samples from patients with CD were collected within 19 months. Only samples within the 19‐month stability range for IL‐8, IFN‐γ, and TNF‐α were reported.

Serum 4β‐hydroxycholesterol and cholesterol were tested using a validated liquid chromatography‐tandem mass spectrometry assay. The internal standards used for 4β‐hydroxycholesterol and cholesterol assays were 4β‐hydroxycholesterol‐d7 and cholesterol‐d7, respectively, with concentration ranges of 2.00‐300 and 0.75‐3.50 mg/mL, respectively. Assay standard and quality control samples were prepared in a surrogate matrix composed of 4% bovine serum albumin in 10 mM phosphate‐buffered saline.

For 4β‐hydroxycholesterol, the mass spectrometry (MS) system consisted of a Sciex API 6500 utilizing turbo ion spray ionization in positive mode, with analyte derivatization and processing using liquid‐liquid extraction. This extract was injected on a Waters BEH C18 1.7‐μm, 2.1 × 150 mm analytical column, and analytes were eluted using gradient elution with a combination of mobile phase A (800:195:5 methanol/water/1 M ammonium acetate) and mobile phase B (1000:5 methanol/1 M ammonium acetate). Monitored MS transitions were *m/z* 402.4 to 385.4 for 4β‐hydroxycholesterol and *m/z* 409.4 to 392.4 for 4β‐hydroxycholesterol‐d7. Assay performance data were: interrun coefficient of variation (CV), ≤14.2%; interrun relative error (RE), –4.5% to 10.7%; intrarun CV, ≤18.0%; intrarun RE, –14.8% to 18.0%; with acceptance criteria of 20%/25% at the lower limit of quantification (LLOQ).

For cholesterol, the MS system consisted of a Sciex API 4000 utilizing turbo ion spray ionization in positive mode, with analytes derivatized and then processed using liquid‐liquid extraction. The extract was injected on a Waters HSS T3 1.8‐μm, 2.1 × 50 mm analytical column, and analytes were eluted using gradient elution with a combination of mobile phase A (5 mM ammonium acetate in 80:20 methanol/water) and mobile phase (5 mM ammonium acetate in methanol). Monitored MS transitions were *m/z* 492.4 to 369.4 for cholesterol and *m/z* 499.4 to 376.4 for cholesterol‐d7. Assay performance data were interrun CV, ≤10.9%; interrun RE, 0% to 11.7%; intrarun CV, ≤11.9%; intrarun RE, –9.9% to 16.2%; with acceptance criteria of 20%/25% at the LLOQ.

Both analytes were stable for at least 24 months in plasma. Samples from patients with UC or CD, and commercially procured samples from healthy subjects were analyzed using the same assays.

All reported concentration data were descriptively summarized by disease status (UC, CD, or healthy) and by treatment period. Data below the lower limit of quantification were imputed as half the LLOQ and were included in the summary, whereas data above the upper limit of quantification were excluded from the summary. Box plots overlaid with individual scatter points were generated to visualize the distribution of individual observations across different groups. For cytokines, scatter points were labeled differently according to sample expiry to assess potential interference with the analysis.

Prednisone, a weak CYP3A inducer,^21^, ^22^ is frequently used as a concomitant medication in patients with IBD. Concomitant use of vedolizumab with other drugs that modulate CYP3A activity may potentially confound results. To rule out any confounding effects of prednisone, its use in VISIBLE 1 and VISIBLE 2 studies, defined as any use of prednisone from 14 days before vedolizumab treatment through the end of treatment, was assessed as a sensitivity analysis. Specifically, spaghetti plots of the 4β‐OHC/C ratio for each patient across the different treatment periods were stratified according to prednisone use to evaluate the presence of any systemic trends.

### Postmarketing Experience

The Takeda global safety database was searched for all reports of DDDI coincident with vedolizumab that were received cumulatively from international birth date (May 20, 2014) to data lock point (May 19, 2020). The high‐level term “interactions” (MedDRA version 23.0), which consists of 19 preferred terms (PTs), as shown in Supplemental Table S1, was used to retrieve the cases. The narratives of these cases were medically reviewed by a single reviewer for any evidence of true DDDIs. All available information including medical history, concomitant medications, therapy details, clinical course of adverse events, and laboratory testing were evaluated to determine if there was strong evidence to establish a true DDDI between vedolizumab and other co–suspect drugs that were reported. Based on the available information, the cases were further categorized into either having limited information or having confounding factors, such as medical history, underlying diseases, and concomitant medications.

## Results

### Literature Data Review

#### Cytokine Concentrations

Concentrations of pro‐ and anti‐inflammatory cytokines in patients with UC or CD compared with those in healthy subjects have been reported in multiple publications.^23^, ^24^, ^25^, ^26^, ^27^, ^28^ As shown in Table 1, the actual cytokine concentrations between groups of patients and heathy subjects varied across different studies. Within most of these studies, patients with UC or CD had elevated serum cytokine concentrations relative to healthy subjects. However, these concentrations were below published concentrations reported to be associated with changes in CYP expression (50‐500 pg/mL).^1^ There were a few instances in which cytokine concentrations were higher than 50 pg/mL, notably IL‐8 and TNF‐α; however, these higher concentrations were observed in both patients with UC or CD and healthy subjects.^25^

**Table 1:** Literature Review Data on Baseline Concentrations (pg/mL) of Serum Cytokines in Patients With UC or CD and Healthy Subjects

Study		Patients With UC or CD						Healthy Subjects					
		n	IL‐1β	IL‐6	IL‐8	IL‐10	TNF‐α	n	IL‐1β	IL‐6	IL‐8	IL‐10	TNF‐α
UC	Holtkamp et al^22^, ^a^	15	NR	10 ± 4^b^	NR	NR	NR	25	NR	7.3 ± 1.2	NR	NR	NR
	Szkaradkiewicz et al^23^, ^c^	20	1.35 ± 1.21^b^	8.63 ± 2.14^b^	31.84 ± 12.97^b^	4.40 ± 1.55^b^	1.18 ± 0.73^b^	15	0.12 ± 0.10	1.59 ± 0.90	9.77 ± 7.65	1.35 ± 0.96	0.61 ± 0.28
	Martinez‐Fierro et al^24^, ^d^	23	3.8 ± 1.0	14.4 ± 3.4	97.9 ± 36.8	15.0 ± 15.8	97.0 ± 26.0	19	3.4 ± 0.5	14.4 ± 10.7	94.9 ± 38.8	15.2 ± 17.4	88.9 ± 22.6
	Korolkova et al,^25^, ^d^ median (IQR)	25	NR	0 (0‐1.49)^b^	6.64 (4.63‐12.51)^b^	NR	7.59 (5.34‐10.16)^b^	30	NR	0 (0‐0.97)	1.93 (1.22‐5.40)	NR	4.3 (2.89‐5.85)
	Ciecko‐Michalska et al,^26^, ^e^ mean (IQR)	35	NR	19.6 (21)^b^	NR	14.4 (5.9)^b^	14.3 (12.6)^b^	35	NR	3.2 (1.6)	NR	3.3 (2.5)	3.1 (1.7)
	Biesiada et al^27^, ^c^	50	3.06 ± 0.27^b^	8.03 ± 0.70^b^	NR	NR	0.96 ± 0.06^b^	NR^f^	1.47 ± 0.12	5.13 ± 0.40	NR	NR	0.62 ± 0.04
CD	Holtkamp et al^22^, ^a^	28	NR	36 ± 8^b^	NR	NR	NR	25	NR	7.3 ± 1.2	NR	NR	NR
	Szkaradkiewicz et al^23^, ^c^	12	0.94 ± 0.84^b^	8.24 ± 1.75^b^	53.70 ± 41.52^b^	2.16 ± 1.46	3.12 ± 2.42^b^	15	0.12 ± 0.10	1.59 ± 0.90	9.77 ± 7.65	1.35 ± 0.96	0.61 ± 0.28
	Martinez‐Fierro et al^24^, ^d^	11	3.4 ± 0.5	18.1 ± 10.6	82.0 ± 16.8	14.9 ± 9.1	93.3 ± 17.2	19	3.4 ± 0.5	14.4 ± 10.7	94.9 ± 38.8	15.2 ± 17.4	88.9 ± 22.6
	Korolkova et al,^25^, ^d^ median (IQR)	28	NR	1.53 (0‐4.85)^b^	4.42 (2.58‐10.93)^b^	NR	5.97 (3.35‐10.34)^b^	30	NR	0 (0‐0.97)	1.93 (1.22‐5.40)	NR	4.3 (2.89‐5.85)
	Ciecko‐Michalska et al,^26^, ^e^ mean (IQR)	39	NR	10.8 (7.6)^b^	NR	10.4 (9.3)^b^	12.6 (11.9)^b^	35	NR	3.2 (1.6)	NR	3.3 (2.5)	3.1 (1.7)

CD, Crohn's disease; IL, interleukin; NR, not reported; SD, standard deviation; TNF, tumor necrosis factor; UC, ulcerative colitis.

All values reported are mean ± SD, unless otherwise specified.

^a^ Cytokine concentrations were measured using immunoradiometric assay.

^b^ Results with statistically significant difference between the patients and healthy controls.

^c^ Cytokine concentrations were measured using enzyme‐linked immunosorbent assay.

^d^ Cytokine concentrations were measured using magnetic‐bead‐based immunoassay.

^e^ The immunoassay used to measure serum concentrations was not specified in the literature.

^f^ Samples were taken from patients with UC in remission; n was not specified in the literature.

#### Exposure to Conventional Medications

Comparable exposure in literature was reported following administration of conventional nonbiologic UC and CD medications metabolized via CYP3A between patients with UC or CD and healthy subjects (or patients with inactive IBD or in remission).

In a study by Milsap et al, the mean AUC ± standard deviation (SD) for intravenous prednisolone did not differ significantly between patients with active UC or CD and those in remission (2516 ± 760 vs 2541 ± 475 ng·h/mL). The authors also concluded that there was no significant difference for the time to peak prednisolone plasma concentration between the 2 groups (1.7 ± 0.6 vs 1.1 ± 0.7 hours).^29^ Following administration of intravenous budesonide in a separate study by Edsbacker et al, the mean AUC (95% confidence interval) was not greater in patients with CD (n = 6) compared with healthy subjects (n = 8): 8.31 nmol·h/L (8.18‐8.44 nmol·h/L) versus 15.1 nmol·h/L (12.5‐18.4 nmol·h/L),^30^ which suggests lack of any suppression of CYP enzymes.

Fluckiger et al conducted a study for oral cyclosporine in which they demonstrated that there was no significant difference between mean ± SD C_max_ or mean ± SD AUC of 19 patients with CD and 23 healthy subjects (C_max_, 864 ± 251 vs 977 ± 348 ng/mL; AUC, 4611 ± 1393 vs 4635 ± 1299 ng·h/mL).^31^ Consistently, it was shown in another study by Schwab et al that the distribution and elimination kinetics of cyclosporine in 12 patients with CD were comparable to healthy subjects after single intravenous (median, 0.8 mg/kg) and oral (median, 3.0 mg/kg) administration.^32^

#### Cytokines and Endogenous CYP3A4 Biomarker Analysis

A total of 182 IBD serum samples (54 samples from 28 patients with UC from VISIBLE 1 and 128 samples from 59 patients with CD from VISIBLE 2) and 40 samples from healthy subjects were included in the analysis (Table 2).

**Table 2:** Patient Disposition in the Retrospective Cytokine and Endogenous CYP3A4 Biomarker Analysis

	Number of Samples				
Analyte	Baseline	Induction^a^	Maintenance^b^—All	Maintenance—Vedolizumab	Maintenance—Placebo
UC (n = 28)	20	18	16	9	7
CD (n = 59)	50	46	32	26	6

CD, Crohn's disease; CYP, cytochrome P450; UC, ulcerative colitis.

Maintenance—All included patients who were randomized midstudy to receive placebo and those who received vedolizumab throughout the duration of the study.

Maintenance—Vedolizumab included only patients who received active vedolizumab treatment throughout the duration of the study.

^a^ Samples were collected in week 6 or 7.

^b^ Samples were collected in week 51 or 52.

#### Cytokine Concentrations

**Figure 1:**
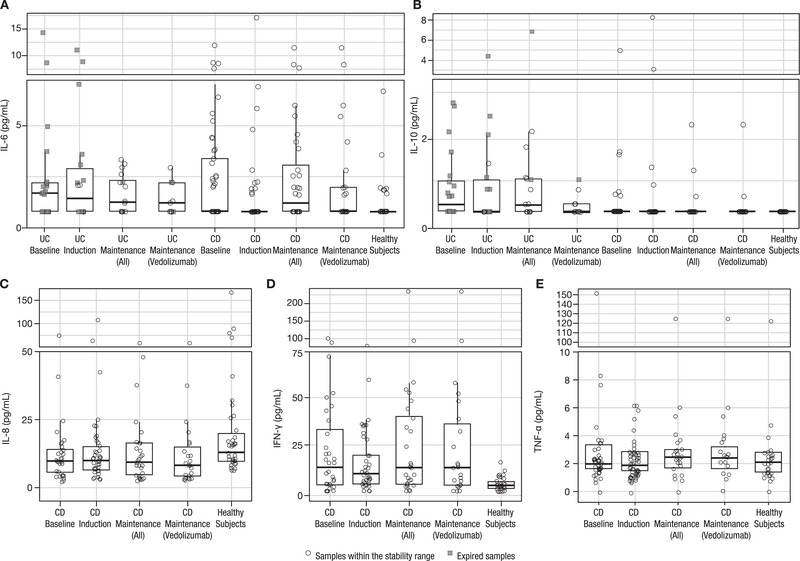
Cytokine concentrations in patients with UC or CD and healthy subjects. (A) Interleukin‐6. (B) Interleukin‐10. (C) Interleukin‐8. (D) Interferon‐γ. (E) Tumor necrosis factor‐α. Interleukin‐1β concentrations were not shown because most samples were below the limit of quantification. Horizontal lines within the box represent median values, the ends of the boxes represent the 25th and 75th percentiles, and the whiskers represent 1.5 times the interquartile range. CD, Crohn's disease; IFN‐γ, interferon gamma; IL, interleukin, TNF‐α, tumor necrosis factor α; UC, ulcerative colitis.

In general, data from vedolizumab trial samples showed no trend for higher cytokine concentrations in patients with UC or CD at baseline relative to healthy subjects except for IFN‐γ, which was used as a reference cytokine to validate the assay platform and to verify patient active disease status (Tables 3 and 4; Figure 1). Although the average concentrations of IL‐6, IL‐10, and TNF‐α were slightly higher in patients with UC or CD compared with healthy subjects, the range of individual data was entirely overlapping across populations. In addition, cytokine concentrations did not change from baseline in response to treatment with vedolizumab during the induction and maintenance phases. The majority of samples had cytokine concentrations within a relatively low concentration of <50 pg/mL, which has been reported in the literature to have no impact on CYP enzymes.^1^ There was no discernible difference between the expired samples and samples within the proven stability interval of 24 months for IL‐6 and IL‐10 (Figure 1).

**Table 3:** Concentrations of Cytokines With 24‐Month Expiry in Patients With UC or CD and Healthy Subjects

		UC				CD				
Analyte	Parameter	Baseline (n = 20)	Induction (n = 18)	Maintenance—All (n = 16)	Maintenance—Vedolizumab (n = 9)	Baseline (n = 49)^a^	Induction (n = 45)^a^	Maintenance—All (n = 32)	Maintenance—Vedolizumab (n = 26)	Healthy Subjects (n = 40)
IL‐6	N_BLQ_ [Link], a	8	9	7	4	28	34	16	16	32
	Mean (SD), pg/mL	2.70 (3.34)	2.78 (3.09)	1.69 (0.971)	1.48 (0.817)	2.52 (2.66)	1.71 (2.71)	2.57 (2.68)	2.27 (2.74)	1.19 (1.07)
IL‐10	N_BLQ_ b, d	10	11	7	5	43	41	29	24	40
	Mean (SD), pg/mL	0.898 (0.806)	0.941 (1.08)	1.14 (1.62)	0.514 (0.264)	0.512 (0.705)	0.613 (1.24)	0.441 (0.381)	0.429 (0.388)	0.340 (0.00)
IL‐1β	N_BLQ_ ^b^, ^e^	20	17	16	9	49	45	32	26	39
	Mean (SD), pg/mL	0.540 (0.00)	0.573 (0.141)	0.540 (0.00)	0.540 (0.00)	0.540 (0.00)	0.540 (0.00)	0.540 (0.00)	0.540 (0.00)	0.768 (1.44)

BLQ, below limit of quantification; CD, Crohn's disease; IL, interleukin; LLOQ, lower limit of quantification; SD, standard deviation; UC, ulcerative colitis.

Most samples from patients with UC were expired (older than 24 months) but were still reported because no decay was observed with stability testing up to 24 months; no samples from patients with CD exceeded 24 months.

^a^ Test was not performed in 1 sample because of insufficient sample volume.

^b^ BLQ imputed to one‐half the LLOQ for analysis.

^c^ LLOQ = 1.62 pg/mL.

^d^ LLOQ = 0.680 pg/mL.

^e^ LLOQ = 1.08 pg/mL.

**Table 4:** Concentrations of Cytokines With 19‐Month Expiry in Patients With CD and Healthy Subjects

		CD				
Analyte	Parameter	Baseline (n = 32)^a^	Induction (n = 42)^a^	Maintenance—All (n = 28)	Maintenance—Vedolizumab (n = 23)	Healthy Subjects (n = 39)^b^
IL‐8	N_BLQ_ ^c^, ^d^	0	0	0	0	0
	Mean (SD), pg/mL	13.0 (13.5)	15.0 (18.2)	13.8 (13.6)	12.5 (13.1)	23.6 (30.4)
IFN‐γ	N_BLQ_ ^c^, ^e^	4	3	3	3	7
	Mean (SD), pg/mL	23.0 (26.2)	16.0 (16.6)	30.1 (46.2)	30.2 (50.3)	4.74 (3.35)
TNF‐α	N_BLQ_ ^c^, ^f^	1	1	1	1	1
	Mean (SD), pg/mL	7.20 (26.4)	2.49 (1.34)	7.00 (22.9)	7.97 (25.3)	5.23 (19.1)

BLQ, below limit of quantification; CD, Crohn's disease; IFN, interferon; IL, interleukin; LLOQ, lower limit of quantification; SD, standard deviation; TNF, tumor necrosis factor.

All baseline and induction samples from patients with UC were expired (older than 19 months) and were not reported because decay was observed with stability testing beyond 19 months. Most samples from patients with CD were collected within 19 months, and only unexpired samples are presented.

^a^ Test was not performed in 1 sample because of insufficient sample volume.

^b^ Results from 1 sample for IL‐8 and from 1 sample for IFN‐γ and TNF‐α were not reported because the sample was above the upper limit of quantification and had inconsistent results, respectively.

^c^ BLQ imputed to one‐half the LLOQ for analysis.

^d^ LLOQ = 1.14 pg/mL.

^e^ LLOQ = 2.60 pg/mL.

^f^ LLOQ = 0.680 pg/mL.

### Endogenous CYP3A Activity

**Figure 2:**
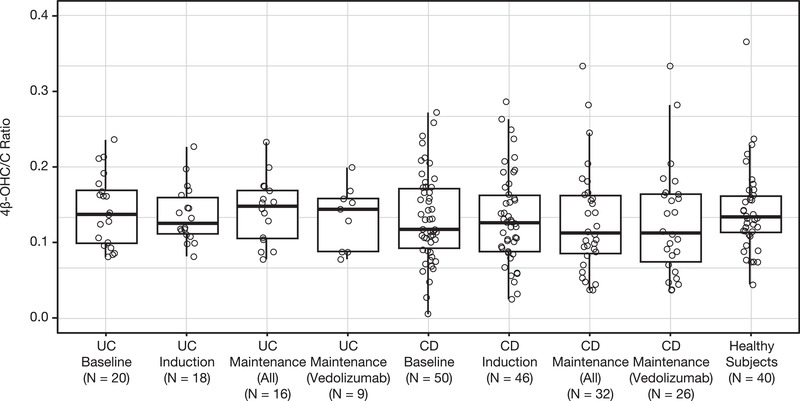
Ratio of 4β‐hydroxycholesterol/cholesterol in patients with UC or CD and healthy subjects. Horizontal lines within the box represent median values, the ends of the boxes represent the 25th and 75th percentiles, and the whiskers represent 1.5 times the interquartile range. 4β‐OHC/C, 4β‐hydroxycholesterol/cholesterol; CD, Crohn's disease; UC, ulcerative colitis.

Data from all samples (UC, 54; CD, 128; healthy subjects, 40) were included for the determination of cholesterol and 4β‐hydroxycholesterol. There was no discernible difference in 4β‐OHC/C ratios between healthy subjects and patients with UC or CD, both before and during vedolizumab treatment (Figure 2), demonstrating that CYP3A4 activity was not modulated by UC or CD disease and/or vedolizumab treatment. For patients with UC, mean ± SD 4β‐OHC/C ratios were 0.143 ± 0.0474 at baseline, 0.137 ± 0.0377 during the induction phase, and 0.143 ± 0.0431 during the maintenance phase of vedolizumab treatment (data not shown). For patients with CD, mean ± SD 4β‐OHC/C ratios were 0.132 ± 0.0581, 0.132 ± 0.0606, and 0.129 ± 0.0699 at baseline, during the induction phase, and during the maintenance phase, respectively. Healthy subjects had a mean ± SD 4β‐OHC/C ratio of 0.142 ± 0.0566.

**Figure 3:**
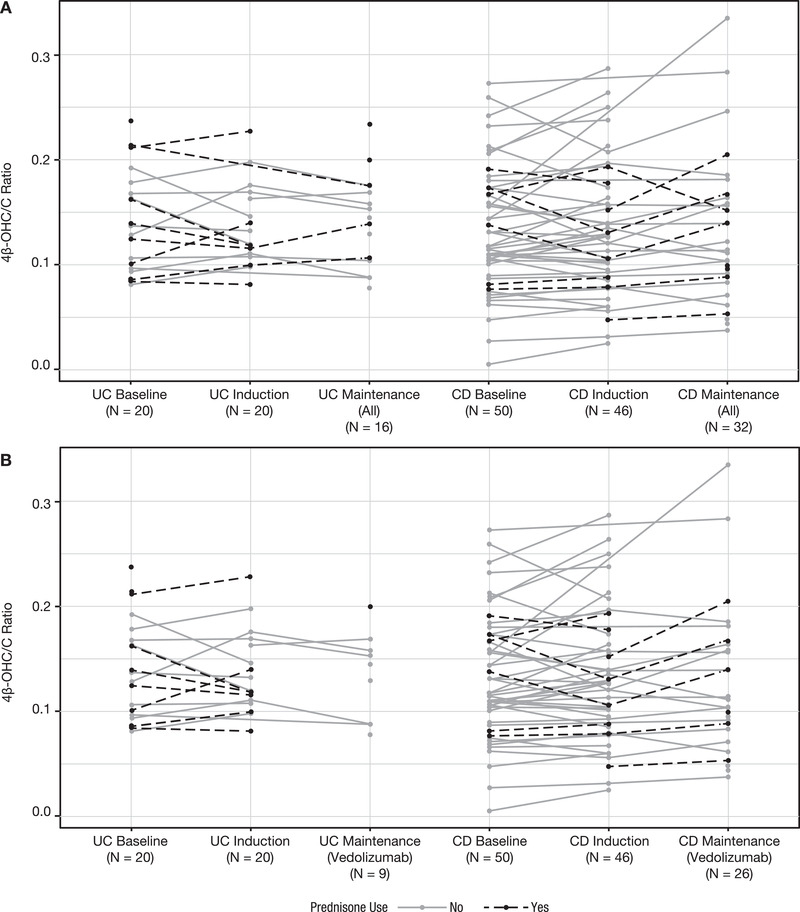
Ratio of 4β‐hydroxycholesterol/cholesterol in patients with UC or CD according to concomitant prednisone use. (A) All patients. (B) Patients receiving vedolizumab in maintenance phase. 4β‐OHC/C, 4β‐hydroxycholesterol/cholesterol; CD, Crohn's disease; UC, ulcerative colitis.

There was also no discernible difference in the 4β‐OHC/C ratios between patients who received concomitant prednisone and those who did not (Figure 3), suggesting that concomitant use of prednisone had no confounding effect on the current evaluation.

### Postmarketing Experience

Using the high‐level term “interactions” (MedDRA version 22.0), only 26 of 191,034 adverse events were reported as suspected DDDIs in the Takeda global safety database search as of the data lock point May 19, 2020.

Most of the events (n = 21) were nonserious, and 5 events were serious. One case reported a PT of “alcohol interaction,” and 25 cases reported a PT of “drug interaction.” Of these 25 cases, 1 case did not involve vedolizumab. Medical review of the remaining 24 cases did not reveal any new safety concerns related to DDDIs: 10 cases reported limited information, in which the details of the medical history, concurrent conditions, concomitant medications, and suspected drug and event details of DDDIs were not reported; 2 cases reported a flare of disease, which is part of the natural course of underlying IBD; 3 cases had leukocytopenia and increased risk of infections, which was attributed to the co–suspect drug azathioprine; 1 case had elevated liver enzymes, which was attributed to another co–suspect drug, Provigil; and 8 cases were confounded by medical history, concurrent conditions, and concomitant medications.

## Discussion

With the increasing clinical use of biologic therapies such as anti‐TNF agents for the treatment of inflammatory diseases,^33^ there has been a growing interest from regulatory agencies and the clinical community in assessing the risk of DDDIs.^10^ The overexpression or activation of proinflammatory cytokines in inflammatory disease states leads to suppression of drug‐metabolizing CYP enzymes, resulting in increased drug concentrations.^3^, ^4^ Several biologic agents have been reported to influence the disposition of concomitant therapies that are CYP substrates because of changes in complex cytokine networks.^2^, ^7^ Treatment of inflammatory diseases with biologics can then reverse the suppression of CYP enzymes, leading to increased metabolism and elimination of these coadministered drugs.^3^ Therefore, evaluation of biologics, such as the anti‐integrin vedolizumab, for indications of inflammatory diseases including IBD is recommended to determine their potential to affect CYP substrates through cytokine modulation.

A clinically significant impact of anti‐inflammatory mAbs on CYP enzyme substrates has been reported, but mainly for cytokine modulators used for the treatment of rheumatoid arthritis (RA). Tocilizumab, an anti‐IL‐6 mAb, was shown to reduce the exposure of simvastatin (CYP3A substrate) by more than 2‐fold in patients with RA.^34^ Therefore, drugs metabolized by CYP3A should be monitored 2 weeks after initiating tocilizumab and 8 weeks after discontinuing treatment, and patients may also require dose adjustments if clinically indicated. In a cocktail DDDI study of sirukumab, another anti‐IL‐6 mAb, exposure of the CYP probe substrates midazolam (CYP3A), omeprazole (CYP2C19), and S‐warfarin (CYP2C9) was reduced by 30% to 35%, 37% to 45%, and 18% to 19%, respectively, and caffeine (CYP1A2) was increased by 20% to 34%, after sirukumab administration in patients with active RA.^35^ However, tildrakizumab, an anti‐IL‐23 mAb, had no clinically relevant effect on the exposure of any CYP probe substrates tested in the cocktail study (S‐warfarin, midazolam, dextromethorphan [CYP2D6], omeprazole, and caffeine) in patients with moderate to severe psoriasis.^36^

Clinical evaluation of DDDIs can be conducted with a cocktail study, which includes simultaneous administration of multiple CYP substrates as probes to assess the drug's potential to inhibit or induce multiple CYP enzymes.^37^ Although cocktail studies provide definitive assessments for DDDIs, the results are limited only to the probe substrates included. In addition, cocktail studies can also be challenging to conduct because of possible operational difficulties such as enrollment (eg, recruitment of patients with high inflammatory burden) and implementation and extra burden to patients, especially in a postmarketing setting.

The stepwise approach utilized in the present study for vedolizumab was based on the recommendations developed at the IQ Consortium/Food and Drug Administration (FDA) workshop (San Diego, California, 2012), which proposed to investigate the following to determine the necessity for a dedicated clinical DDDI study: effect of the disease on cytokine concentrations and CYP expression, mechanism of action of the drug and its impact on cytokine regulation, risks related to concomitant medication and clearance mechanisms, and clinical approaches in determining DDI risk on CYP enzymes.^10^

Cytokine concentrations in patients with IBD have been reported in multiple publications.^23^, ^24^, ^25^, ^26^, ^27^, ^28^ The high degree of variability in the reported concentrations of these cytokines across different studies may likely be attributed to the different bioassay methods used. However, because samples from both patients and healthy subjects were measured using the same assay within each individual study summarized in Table 1, a cross‐population comparison within each study is still reasonable. This literature review suggests that cytokine concentrations were comparable between patients with IBD and healthy subjects and/or remained at a low concentration that is unlikely to cause any change in CYP enzymes. This is consistent with the results of our current study based on samples from both patients with UC or CD and healthy subjects analyzed using the same assay. Only the concentration of IFN‐γ, which is considered one of the most highly upregulated cytokines in UC and CD,^19^ was noted to be higher in patients with UC or CD compared with healthy subjects, but concentrations were still below the reported concentrations that could impact CYP expression. The number of subjects included in our current study is comparable to that in published studies, and the level of assay variability is also generally comparable to or less than that reported in the literature.

The effect of vedolizumab on cytokines has been evaluated in isolated human leukocytes in vitro and in clinical studies. Binding of vedolizumab to peripheral blood lymphocytes did not elicit cytokine production, including IFN‐γ, TNF‐α, IL‐1β, IL‐2, IL‐4, IL‐6, IL‐8, IL‐12 (p70), and IL‐17.^38^ As vedolizumab is gut‐selective and not a cytokine modulator,^12^ the potential for CYP enzyme‐mediated DDDIs is considered low. This was confirmed by the lack of change in cytokine concentrations and 4β‐OHC/C ratio before and after vedolizumab treatment in our current study. The 2 identified confounding factors, sample expiry and concomitant use of prednisone, which were evaluated in the analysis of cytokines and 4β‐OHC/C, respectively, showed no visible trend. Prednisone was reported to be a weak CYP3A inducer in some articles in the literature.^21^, ^22^ Prednisone only marginally activates the receptor responsible for the induction of CYP3A, with a maximum activation of 10.7‐fold versus 100‐fold with rifampicin, which is a potent CYP3A inducer.^39^ Coadministration of prednisone was also shown to have no clinically meaningful effect on the pharmacokinetics of CYP3A substrates.^40^, ^41^, ^42^ Therefore, prednisone use was not expected to interfere with the current evaluation.

Utilization of an endogenous biomarker for CYP3A activity is a preferred method to assess DDDIs from a safety perspective.^43^, ^44^ Administration of exogenous probe substrates is avoided, and results can be based on only a single blood sample at any point, which is a more feasible assessment, especially in patients with active disease status. Therefore, the identification of endogenous biomarkers has diminished the need for a dedicated clinical DDDI study. Of note, the use of 4β‐OHC and 4β‐OHC/C as endogenous biomarkers has been demonstrated previously, with 4β‐OHC concentration reported to increase and decrease after treatment with CYP3A inducers and inhibitors, respectively.^45^, ^46^

However, these biomarkers do have some limitations. Intraindividual variability of 4β‐OHC over time has been shown to be 7.1%^47^ and variability between patients to be 120‐fold.^46^ Factors that can contribute to this variability include genetics, sex, disease, and comedication with CYP3A inducers or inhibitors.^46^, ^48^, ^49^ Prediction of induction and inhibition activities with 4β‐OHC was also reported to be less sensitive than with oral midazolam, a well‐established exogenous CYP3A probe substrate.^44^ After administration of ketoconazole, a potent CYP3A inhibitor, and rifampicin, a potent CYP3A inducer, mean 4β‐OHC values decreased up to 23% and increased up to 228%, respectively, whereas mean midazolam AUC increased by 11‐fold and decreased by at least 95%, respectively.^44^ Furthermore, correlation of 4β‐OHC/C ratio with systemic and oral midazolam clearance (Spearman's rank correlation coefficient [*r*_s_] = 0.348 and *r*_s_ = 0.353, respectively) was weak.^50^ The 4β‐OHC/C ratio may be more appropriate as an endogenous CYP3A marker rather than 4β‐OHC alone, as the 4β‐OHC/C ratio accounts for individual variations in plasma cholesterol concentration.^20^, ^48^, ^51^ It should be noted, however, that decreased CYP3A activity may only be evident if the effects of the administered CYP3A inhibitors are sufficiently strong.^50^ In addition, these endogenous biomarkers would have been generally more valuable in a crossover study design, in which each patient serves as his/her own control to minimize intraindividual variability.^7^ However, because the DDDI potential between biologic agents and small‐molecule CYP substrates mainly arises from disease effect, and given that biologic agents typically have long half‐lives, evaluation of DDDI for biologics generally cannot be performed in a crossover study, thus limiting the value of using endogenous biomarkers.

Both VISIBLE 1 and VISIBLE 2 were conducted recently with large sample sizes; hence, a reasonable number of samples within the stability range of the analytes were included in the present study. Vedolizumab exposure achieved with subcutaneous administration (108 mg every 2 weeks) was in a similar range to intravenous administration (300 mg every 8 weeks). Furthermore, vedolizumab subcutaneously was shown to have similar efficacy and safety profiles to vedolizumab intravenously. Therefore, the conclusion of vedolizumab treatment having no effect on cytokine and CYP3A4 biomarker concentrations should be applicable to both subcutaneous and intravenous formulations of vedolizumab. Medical review of reported DDDI cases from the postmarketing data also suggested a minimal DDDI risk for vedolizumab, supporting its relevance as a long‐term therapy for patients with UC or CD.

## Conclusions

In conclusion, this study found that IBD neither meaningfully affects serum concentrations of cytokines known to impact CYP expression nor influences the exposure of CYP substrate drugs. Vedolizumab treatment also did not affect cytokine concentrations or CYP3A activity in patients with moderately to severely active UC or CD. These results confirm that there is minimal risk of DDDI in IBD patients treated with vedolizumab, adding further evidence to the established safety profile of vedolizumab in IBD.

## Conflicts of Interest

Wan Sun, Jiri Aubrecht, and Maria Rosario were employees of Takeda at the time this research was conducted. Richard A. Lirio, Harisha Kadali, Mike Baratta, Parul Gulati, Ajit Suri, and Raghavan Vasudevan are employees of Takeda and hold stock or stock options. Jennifer Schneider and Tiffany Lin are employees of Certara USA, which received funding from Takeda.

## Funding

This study was sponsored by Takeda.

## Data‐Sharing Statement

The data sets, including the redacted study protocol, redacted statistical analysis plan, and individual participants data supporting the results reported in this article, will be made available within 3 months from initial request to researchers who provide a methodologically sound proposal. The data will be provided after its deidentification, in compliance with applicable privacy laws, data protection, and requirements for consent and anonymization.

## Author Contributions

All authors contributed to the study concept and design and were involved in interpreting the data. All authors contributed to the drafting or revising of the article and gave their final approval of the version to be published.

## Supporting information

Supporting Information
