# Supplementary material for: Assessment of Vedolizumab Disease‐Drug‐Drug Interaction Potential in Patients With Inflammatory Bowel Diseases
Source: Clin Pharmacol Drug Dev. 2020 Dec 16;10(7):734–47. doi: 10.1002/cpdd.891 (PMC8359401; doi:10.1002/cpdd.891)
Supplement: Supplementary file 1 — Supporting Information [file CPDD-10-734-s001.docx]

**Supplemental Material**

**Supplemental Table S1. Preferred Terms under the MedDRA High-Level Term “Interactions” used in the Takeda global safety database search**

| **Term** |
| --- |
| Alcohol interaction  Device interaction  Drug chemical incompatibility  Drug interaction  Drug physiologic incompatibility  Drug therapeutic incompatibility  Drug-device interaction  Drug-disease interaction  Food interaction  Herbal interaction  Inhibitory drug interaction  Labelled drug-disease interaction medication error  Labelled drug-drug interaction medication error  Labelled drug-food interaction medication error  Potentiating drug interaction  Radiation interaction  Therapeutic agent-diagnostic test interaction  Tobacco interaction  Tyramine interaction |
